# Exosomes in cartilage microenvironment regulation and cartilage repair

**DOI:** 10.3389/fcell.2025.1460416

**Published:** 2025-03-05

**Authors:** Han Longfei, Hou Wenyuan, Fang Weihua, Peng Peng, Lu Sun, Lin Kun, He Mincong, Yang Fan, He Wei, Wei Qiushi

**Affiliations:** ^1^ Guangzhou University of Chinese Medicine, Guangzhou, Guangdong, China; ^2^ Traumatology and Orthopedics Institute of Guangzhou University of Chinese Medicine, Guangzhou, Guangdong, China; ^3^ Department of Orthopaedics, The Third Affiliated Hospital of Guangzhou University of Chinese Medicine, Guangzhou, Guangdong, China; ^4^ State Key Laboratory of Traditional Chinese Medicine Syndrome/Orthopaedic, The Third Affiliated Hospital of Guangzhou University of Chinese Medicine, Guangzhou, Guangdong, China

**Keywords:** exosomes, osteoarthritis, cartilage repair, cartilage microenvironment, mechanism of action

## Abstract

Osteoarthritis (OA) is a debilitating disease that predominantly impacts the hip, hand, and knee joints. Its pathology is defined by the progressive degradation of articular cartilage, formation of bone spurs, and synovial inflammation, resulting in pain, joint function limitations, and substantial societal and familial burdens. Current treatment strategies primarily target pain alleviation, yet improved interventions addressing the underlying disease pathology are scarce. Recently, exosomes have emerged as a subject of growing interest in OA therapy. Numerous studies have investigated exosomes to offer promising therapeutic approaches for OA through diverse *in vivo* and *in vitro* models, elucidating the mechanisms by which exosomes from various cell sources modulate the cartilage microenvironment and promote cartilage repair. Preclinical investigations have demonstrated the regulatory effects of exosomes originating from human cells, including mesenchymal stem cells (MSC), synovial fibroblasts, chondrocytes, macrophages, and exosomes derived from Chinese herbal medicines, on the modulation of the cartilage microenvironment and cartilage repair through diverse signaling pathways. Additionally, therapeutic mechanisms encompass cartilage inflammation, degradation of the cartilage matrix, proliferation and migration of chondrocytes, autophagy, apoptosis, and mitigation of oxidative stress. An increasing number of exosome carrier scaffolds are under development. Our review adopts a multidimensional approach to enhance comprehension of the pivotal therapeutic functions exerted by exosomes sourced from diverse cell types in OA. Ultimately, our aim is to pinpoint therapeutic targets capable of regulating the cartilage microenvironment and facilitating cartilage repair in OA.

## 1 Introduction

Osteoarthritis (OA), recognized as the prevailing joint pathology globally, is distinguished by the degradation of articular cartilage, formation of bone redundancy, subchondral bone sclerosis, and inflammation and fibrosis in the synovium, predominantly affecting joints such as the hip, hand, and knee. This degenerative process leads to functional constraints and impairment in joint functionality. Current pharmacological interventions, serving as palliative measures, fail to halt or reverse the relentless advancement of the ailment, culminating in the necessity for joint replacement (Fan et al., 2022). Hence, there exists a pressing imperative to innovate novel therapeutic modalities that not only arrest but also potentially reverse the trajectory of OA. In OA, intricate biomechanical transformations transpire within the cartilage and chondrocytes, involving cartilage deterioration, mechanical stress, and modifications in the composition of the cartilage matrix, mediated by vital agents comprising matrix-degrading enzymes and inflammatory cytokines (Chen L. et al., 2024). While chondrocytes reside in a quiescent state in the pristine joint environment marked by diminished metabolic activity and regulated exchange of matrix constituents, upon injury to articular cartilage, chondrocytes mount a reparative response, paradoxically exacerbating arthritic progression due to constrained vascular supply to the cartilage matrix. Resultantly, an accelerated process of cartilage matrix degradation outpaces the chondrocytic synthesis of new matrix elements (Adam et al., 2024; Lee et al., 2024). Consequently, emphasis on the pivotal role of chondrocytes in the therapeutic paradigm of OA is warranted. Damage to articular cartilage or an inflammatory response accelerates joint degeneration, representing a significant pathological manifestation of OA. The challenges associated with repairing cartilage damage are attributed to the absence of blood supply, innervation, and lymphatic tissue within the joint cavity, compounded by the negative pressure environment resulting from ischemia and hypoxia (Chen J. et al., 2024). Formerly extolled as a promising intervention approach, cell-based therapy has manifested some efficacy through intra-articular instillations of pertinent cells, such as mesenchymal stem cells, showing promise in mitigating inflammatory cascades and alleviating pain (Zelinka et al., 2022; Copp et al., 2023). Conversely, empirical evidence underscores the potential unpredictability of cellular therapeutics, evoking adverse sequelae encompassing joint discomfort, edema, swelling, and even grave ramifications like carcinogenesis (Shang et al., 2023). These limitations are also evident in the loss of grafted cells, cellular senescence, apoptosis, hypertrophy, inflammation, and necrosis (Steinert et al., 2007; Alahdal et al., 2021). Exosomes (Exo) wield paramount significance in intercellular signaling mechanisms, serving as pivotal conduits for biological communication among cellular entities to regulate a myriad of physiological processes. Operating as vital signaling moieties, exosomes orchestrate the transfer of proteins, lipids, and nucleic acids, constituting a significant role in diverse physiopathological processes including tissue restitution and immune surveillance (Xu X. et al., 2024). Capitalizing on the multifaceted functions of exosomes, the manipulation of the cartilage microenvironment and facilitation of cartilage repair hold profound therapeutic promise in tackling OA (Yue et al., 2024). Despite elucidative advancements in delineating the molecular underpinnings by which exosomes foster cartilage regeneration, a comprehensive exploration of the diverse cellular origins of exosomes influencing cartilage repair in OA remains a nascent domain. Therefore, a meticulous review delineating the regulatory efficacy of exosomes derived from distinct cellular reservoirs in orchestrating cartilage healing in OA is warranted to furnish a seminal reference for subsequent investigational pursuits.

## 2 Overview of extracellular vesicles and exosomes

**FIGURE 1:**
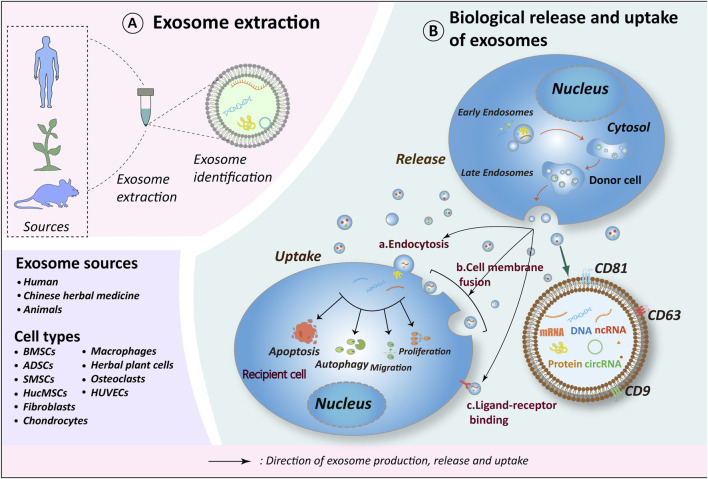
Exosome biorelease and uptake process. **(A)** Exosomes originate from diverse sources, with current research emphasizing those derived from human tissues, plants (including herbs), and animal tissues. The cell types of exosomes exhibiting therapeutic potential for OA include BMSCs, ADSCs, SMSCs, HucMSCs, Fibroblasts, Chondrocytes, Macrophages, Osteoclasts, HUVECs and herbs. **(B)** The biogenesis of exosomes occurs in four distinct stages: 1. Endocytosis, during which the cell membrane invaginates to form early endosomes; 2. Packaging of contents, wherein early endosomes further mature into late endosomes; 3. Fusion of late endosomes to form multivesicular bodies (MVBs); 4. MVBs subsequently fuse with the cell membrane to release their contents into the extracellular compartment, thereby forming exosomes. Recipient cells internalize exosomes *via* endocytosis, direct fusion, or ligand-receptor interactions. Through the release of materials encapsulated within exosomes (e.g., proteins, nucleic acids, lipids, metabolites), exosomes exert influence on the metabolic processes of recipient cells, including cell proliferation, migration, apoptosis, and autophagy, thereby further impacting the OA process. Schematic drawing reference from Chen J. et al. (2022). BMSCs, bone marrow mesenchymal stem cells; ADSCs, adipose mesenchymal stem cells; SMSCs, synovial mesenchymal stem cells; HucMSCs, human umbilical cord mesenchymal stem cells; HUVECs, human umbilical vein endothelial cells.

Extracellular vesicles (EVs) are lipid-membrane vesicles secreted by cells into the extracellular space. They exhibit diameters ranging from 30 nm to 2,000 nm and encompass three subtypes: microvesicles, exosomes, and apoptotic vesicles. EVs comprise approximately 0.1% of the RNA content found in parental cells and exhibit diverse mechanisms through which they influence biological processes. This involves proteins and biologically active lipid ligands present on the vesicle surface mediating membrane fusion by engaging cell surface receptors. Consequently, vesicles release their contents—comprising transcription factors, oncogenes, non-coding RNAs, mRNAs, and infectious particles—to recipient cells, thus modulating their functions (Andaloussi et al., 2013; Lin et al., 2016). Among extracellular vesicles, exosomes are extensively researched and can be emitted by all cell types. These membranous structures have been detected in various bodily fluids, including plasma, urine, saliva, lymph, and synovial fluid (Doyle and Wang, 2019). A schematic diagram of exosomes is shown in Figure 1.

Several techniques exist for the extraction, isolation, and purification of exosomes, albeit lacking uniform standards. Common methodologies encompass differential ultracentrifugation, density gradient centrifugation, kit-based procedures, magnetic bead-based immunoaffinity capture, and size-exclusion chromatography (SEC). The rationale for these solutions is grounded in the unique physical and molecular biological properties of exosomes. While numerous methods for exosome isolation exist, each presents specific limitations (Ma X. et al., 2024). Ultracentrifugation leverages the size differential between exosomes and other components in the cell culture supernatant for effective separation. Although ultracentrifugation is regarded as the gold standard for exosome isolation, it is time-consuming, requires expensive centrifuge equipment, and is prone to contamination by various impurities (e.g., other particles, viruses, lipoprotein particles, and protein complexes). Additionally, it may cause structural disruption of the exosomes, potentially affecting downstream analyses (Yang et al., 2020). These disadvantages further restrict the applicability of ultracentrifugation. In contrast to ultracentrifugation, density gradient centrifugation operates on the principle that different vesicle types exhibit varying densities and sedimentation rates. The two primary methods include optiprep density medium separation and sucrose density medium separation. This method necessitates the use of a density gradient medium. Under high centrifugal force, the particles within the sample migrate at varying rates and remain suspended in the solution without the need for layering. This separation technique is particularly suitable for the purification of large-volume samples. However, this method is time-consuming, cumbersome, and susceptible to sample damage (Zhu et al., 2019). To enhance the specificity and targeting of exosome isolation, the magnetic bead-based immunoaffinity capture technique can be employed (Jiawei et al., 2022). This method employs immunological techniques to specifically recognize and isolate exosomal membrane proteins, such as CD9, CD63, and CD81, resulting in significantly higher purity compared to exosomes isolated solely based on physical properties (Tauro et al., 2012). However, the differential expression of exosomal membrane proteins frequently results in insufficient exosome yield. Size exclusion chromatography separates exosomes based on molecular weight and size and is currently more widely utilized for the removal of mixed proteins and lipids from exosome samples (Gámez-Valero et al., 2016). This method is advantageous for preserving the biological activity of exosomes; however, it necessitates expensive equipment, is time-consuming, and is best suited for processing small sample volumes (Gheinani et al., 2018). Nevertheless, the application of size exclusion chromatography for exosome extraction has garnered increasing attention in recent years (Yang et al., 2020). Notably, differential ultracentrifugation, the pioneering technique for exosome isolation, persists as the preferred method in current exosome research (Doyle and Wang, 2019; Fan et al., 2024; Yue et al., 2024).

## 3 Association of exosomes with OA

Exosomes exert their distinct functions via intercellular signaling, influencing key physiological processes like development, cell proliferation, differentiation, and metabolism. Increasing research indicates the vast potential of exosomes in tissue regeneration, disease therapy across various conditions, and in conferring protective effects to the body (Tang et al., 2014; Chen C. Y. et al., 2022; Wei et al., 2023). Exosomes hold significance in orthopedic degenerative ailments such as OA, osteoporosis, and disc degeneration. Studies have highlighted 46 distinctively expressed miRNAs linked to processes like inflammation, autophagy, chondrocyte viability, differentiation, homeostasis, metabolism, and extracellular matrix degradation in individuals with OA (Cong et al., 2017). Due to their enhanced targeting capacity and simple storage and management feasibility (Li S. et al., 2024), exploring miRNAs further holds promise in elucidating OA pathogenesis and potentially altering the disease trajectory.

OA is therapeutically characterized by a variety of disease processes and pathologies, which complicates the development of universally effective treatments (Hart, 2022). Treatment strategies do not focus solely on a single joint’s pathology; instead, they integrate behavioral, psychosocial, educational, and physical interventions (Kolasinski et al., 2020). Based on the treatment modality, approaches may be categorized into two primary types: non-pharmacological and pharmacological (Kolasinski et al., 2020; Katz et al., 2021). Non-pharmacological interventions, including walking, swimming, tai chi, yoga, cognitive behavioral therapy (CBT), and acupuncture, have been shown to enhance joint function in patients with OA (Bannuru et al., 2019). Conversely, pharmacological approaches primarily consist of non-steroidal anti-inflammatory drugs (NSAIDs) (Conaghan et al., 2008), the development of disease-modifying OA drugs, and regenerative medicine (Cho et al., 2021). Stem cells have been rigorously investigated in tissue regeneration research due to their capacity for redifferentiation and the advantageous components found in their secreted factors. Given the instability of stem cells in the human body, their secreted exosomes are regarded as a safer alternative for the next-generation of cell-based therapies (Alcaraz et al., 2019).

## 4 Exosomes originating from various cellular sources

### 4.1 Mesenchymal stem cell-derived exosomes

Mesenchymal stem cells (MSCs), possessing self-renewal and differentiation capabilities, demonstrate significant potential in OA therapy. Serving as pivotal cells in regenerative approaches, reports exist on the transplantation of MSCs sourced from diverse origins such as bone marrow, adipose tissue, synovium, blood, and umbilical cord (Wen et al., 2012; Chen et al., 2016; Chen et al., 2014; Fu et al., 2016; Otsuki et al., 2018). In contrast to conventional cell transplantation techniques, extracellular vesicles (EVs) present a safer and innovative avenue for the prevention and treatment of OA. Progressive research findings increasingly indicate that the therapeutic benefits of stem cell transplantation may be facilitated through the actions of their extracellular vesicles (Nguyen et al., 2021; Rizzo et al., 2023). Exosomes from stem cells, such as MSCs, carry essential growth factors and other bioactive molecules that promote tissue repair and regeneration, facilitating healing processes and enhancing cell communication (José, 2024). Hence, there is a critical need to investigate the mechanism by which exosomes sourced from MSCs modulate the cartilage microenvironment and facilitate cartilage restoration.

#### 4.1.1 Bone marrow mesenchymal stem cells -derived exosomes

The Wnt/β-catenin pathway is a well-established signaling cascade linked to bone formation, tissue regeneration, and joint equilibrium, playing a crucial role in the development of OA (Huang et al., 2020; Feng et al., 2024). Activation of the Wnt signaling pathway induces the stabilization and nuclear translocation of β-catenin, thereby further activating target gene expression related to cartilage degradation, inflammation, and bone regrowth (Usami et al., 2016; Aziz et al., 2024). The Wnt signaling pathway is categorized into two modes based on β-catenin dependence: the β-catenin-dependent mode, referred to as the classical Wnt/β-catenin signaling pathway, and its counterpart, known as the non-classical pathway. The classical Wnt pathway is initiated by the binding of Wnt to the Wnt receptor (i.e., Frizzled) and the Wnt co-receptor (i.e., lipoprotein receptor-related protein-LRP-5/6), which subsequently induces conformational changes in the downstream molecular complex. The downstream molecular complex comprises dishevelled, glycogen synthase kinase 3β (GSK3β), axin, adenomatosis polyposis coli (APC), β-catenin, and other associated proteins. Alterations in the interactions and phosphorylation order of constituent proteins disrupt the phosphorylation of the amino-terminal structural domain of β-catenin, leading to its stabilization and nuclear translocation. This process, in turn, stimulates the transcription of target genes in conjunction with T-cell factors and lymphoid enhancer-binding factors (TCF/LEF), among others. Wnt-associated proteins exhibit differential expression in various regions of cartilage (Witte et al., 2009). Notably, Wnt4, Wnt5a, Wnt5b, Wnt11, and Wnt14 are significantly differentially expressed in cartilage, perichondrium, and surrounding tissues (Hartmann and Tabin, 2000). Wnt4 and Wnt14 are predominantly localized in articular cartilage (Hartmann and Tabin, 2001); Wnt5a is highly expressed in cartilage spanning the epiphysis to the metaphysis (Church et al., 2002); Wnt5b is primarily expressed in the prehypertrophic region; and Wnt11 is predominantly found in chondrocytes during embryonic limb development. Notably, the activation of the Wnt/β-catenin pathway must be tightly regulated to facilitate normal cartilage development, as either excessive activation or insufficient activation of this signaling pathway can lead to skeletal diseases (Yuasa et al., 2009; Dao et al., 2012).

Exosomes originating from BMSCs have been shown to enhance cartilage regeneration through the stimulation of chondrocyte proliferation and migration while preventing apoptosis, mainly mediated by the Wnt/β-catenin pathway. Subsequent investigations have identified that the distinctive molecular underpinnings of BMSC-derived exosomes involve the enrichment of miR-127-3p, miR-92a-3p, miR-140-3p, along with proteins associated with cellular adhesion and tissue restoration capabilities (Mao et al., 2018; Dong et al., 2021; Hu Y. et al., 2023; Wang L. et al., 2024).

The PI3K/AKT/mTOR pathway stands as a critical cellular cascade governing cell proliferation, motility, growth, and metabolism, essential for maintaining the homeostasis of joint tissues and implicated in the pathogenesis of OA (Sun et al., 2020). Glucose, insulin, growth factors, and cytokines all activate the PI3K/AKT/mTOR signaling pathway (Engelman et al., 2006). These molecules function by activating receptor tyrosine kinases (RTKs) and G protein-coupled receptors (GPCRs), thereby stimulating PI3K to produce phospholipids and activate downstream proteins, including AKT and the mammalian target of rapamycin complex 1 (mTORC1) (De Santis et al., 2019). Protein kinase B (AKT) serves as a crucial signaling molecule within the PI3K pathway. Once activated, AKT translocates to various cellular compartments to activate several downstream substrates, including protein kinases, E3 ubiquitin ligases, small G-protein modulators, metabolic enzymes, transcription factors, and cell cycle regulators (Manning and Toker, 2017). A key downstream branch of AKT is mTORC1. Phosphorylation of AKT promotes the phosphorylation of mechanistic target of rapamycin kinase (mTOR) at Ser2448 and directly activates mTORC1. Altered mTORC1 activity subsequently affects its effectors, including S6 kinase 1 (S6K1), eukaryotic translation initiation factor 4E binding protein 1 (4EBP1), and unc-51-like kinase 1 (ULK1). Among these, S6K1 and 4EBP1 promote the translation of mRNAs for hypoxia inducible factor 1 subunit alpha (HIF-1α), cyclin D1, and myelocytomatosis oncogene (c-Myc), thereby participating in processes such as cell cycle regulation and angiogenesis (Laplante and Sabatini, 2012). Notably, mTORC1 serves as a major regulator of unc-51 like autophagy activating kinase 1 (ULK1) and is associated with the initiation of autophagy. Treatment with rapamycin, an inhibitor of mTORC1, enhances ULK1 kinase activity; conversely, the promotion of mTORC1 activity effectively inhibits ULK1 activity (Park et al., 2016; Nnah et al., 2019). Furthermore, ULK1 can form a complex by binding to autophagy related 13 (ATG13) and fak family kinase-interacting protein (FIP200), acting as a node that translates autophagic signaling into the initiation of autophagic biological processes (Ganley et al., 2009). These observations suggest that the PI3K/AKT/mTOR signaling pathway is critical for maintaining cellular homeostasis and is closely associated with various biological processes, including the cell cycle, cell survival, inflammation, metabolism, and apoptosis (Cravero et al., 2009; Tang et al., 2018).

Research has revealed that exosomes derived from BMSCs mediate KLF 3-AS 1, activating the PI3K/Akt/mTOR pathway. They also competitively adsorb miR-206 to enhance G protein-coupled receptor kinase interacting ArfGAP 1 (GIT 1) expression, thereby fostering chondrocyte proliferation and inhibiting apoptosis. Consequently, KLF 3-AS 1 emerges as a promising candidate for therapeutic interventions aimed at enhancing articular cartilage repair (Liu et al., 2018a; Wen et al., 2022). Another investigation demonstrated that exosomes derived from BMSCs treated with decellularized extracellular matrix (dECM) enhanced the expression of miR-3473b, mediating the phosphatase and tensin homolog (PTEN)/protein kinase b. (AKT) pathway to ameliorate cartilage damage in a similar manner (Liu et al., 2018b). Additionally, BMSC-derived exosomes exhibit the ability to suppress chondrocyte apoptosis by mediating miR-326 and targeting the histone deacetylase 3 (HDAC3), signal transducer and activator of transcription 1 (STAT1), and nuclear factor kappa b (NF-κB)/p65 signaling pathways (Xu and Xu, 2021).

Mechanical stress plays a crucial role in stimulating cell proliferation and differentiation. Studies have demonstrated that low-intensity pulsed ultrasound (LIPUS) promotes the inhibition of inflammation, enhances chondrocyte proliferation, and stimulates cartilage matrix synthesis through mechanisms closely associated with the NF-κB pathway (Liao et al., 2021). Additionally, pulsed electromagnetic fields (PEMF) at 75 Hz have shown comparable efficacy in these processes (Xu et al., 2023). Furthermore, exosomes derived from BMSCs overexpressing miRNA-210 have shown potential in safeguarding chondrocytes from damage responses in inflammatory conditions via this pathway (He et al., 2020). Besides mechanical factors, proteins influenced by mechanical stress have been linked to cartilage restoration. Research has indicated that the transfection of exosomes from bone marrow endothelial cells (BMECs) with silenced Piezo1 lentivirus not only hampers the inflammatory response but also enhances cartilage repair processes (Li et al., 2021). These findings imply that Piezo1 may hinder OA therapy and holds potential as a target for therapeutic interventions.

Macrophages are the predominant immune cells in synovial tissue involved in inflammation and tissue damage repair processes. Studies have shown that both BMSCs-Exos and HucMSCs-Exos possess the ability to modulate macrophage polarization from the M1 to M2 type (Li X. et al., 2024; Tian et al., 2024). Furthermore, BMSCs-Exos have been found to suppress inflammation and enhance cartilage repair by orchestrating the glutamic-oxaloacetic transaminase 1 (GOT1)/c-c motif chemokine receptor 2 (CCR2) pathway, resulting in increased expression of NF-E2-related factor 2 (Nrf2)/heme oxygenase 1 (HO-1), thereby inhibiting macrophage iron death (Peng et al., 2023). Additionally, MSCs exposed to aging and inflammatory conditions demonstrate reduced effectiveness in alleviating the pathological progression of OA (Jérémy et al., 2024). This indicates that while bone marrow MSCs have the potential to differentiate into cartilage, the cellular microenvironment and physiological conditions are crucial factors influencing the functionality of the exosomes they secrete. Moreover, BMSCs-Exos promote chondrocyte mitochondrial autophagy by regulating dynamin-related protein 1 (Drp1) expression, leading to the inhibition of chondrocyte apoptosis and cartilage matrix degradation (Tang et al., 2021).

Using pharmacological agents known for promoting cartilage regeneration and chondroprotection to treat BMSCs, followed by the extraction of exosomes for studying their effects on chondrocytes, represents valuable research strategies for elucidating drug mechanisms and exploring the therapeutic potential of the exosomal pathway (Wang and Xu, 2021). *In vitro* studies have demonstrated the substantial anti-inflammatory, antioxidant, antidiabetic, and immunomodulatory properties of fucoidan. Particularly noteworthy is the enrichment of miR-146b-5p in fucoidan-treated BMSCs-Exos, which effectively inhibited tumor necrosis factor receptor-associated factor 6 (TRAF 6) activation, leading to the attenuation of inflammatory reactions and preservation of extracellular matrix integrity (Lou et al., 2023). Transforming growth factor-β1 (TGF-β1) is synthesized and secreted by chondrocytes, where it remains latent in the extracellular matrix (ECM) of various tissues before activating and stimulating chondrocytes to produce essential ECM elements. When BMSCs-Exos were exposed to TGF-β1, enhanced cartilage repair efficacy was observed compared to BMSCs-Exos treatment alone. Discrepancies in the levels of miR-373 and miR-483 within their exosomal content were evident, alongside increased expression of miR-135b following TGF-β1 treatment, which in turn downregulated sp1 transcription factor (Sp1), ultimately facilitating cartilage repair (Wang et al., 2018).

In conclusion, the remarkable potential for OA treatment is evident, owing to the exceptional delivery capacity and diverse biological functionalities exhibited by exosomes released by BMSCs.

#### 4.1.2 Adipose-derived mesenchymal stem cells -derived exosomes

Adipose-derived stem cells (ADSCs), owing to their ease of procurement and notable chondrogenic differentiation potential in treating articular cartilage injuries, are categorized based on their origin as either infrapatellar fat pad (IPFP) or subcutaneous fat-derived MSCs. Notably, ADSCs exhibit superior chondrogenic capabilities compared to other MSCs and display resilience to inflammatory influences, thereby attracting significant research focus and scrutiny (Liao et al., 2022).

Kartogenin (KGN) is recognized as a potent stimulator of mesenchymal stem cells for chondrocyte differentiation. Studies have demonstrated that KGN effectively promotes chondrogenic differentiation in synovial MSCs (Xu et al., 2021). Furthermore, in comparative analyses with exosomes derived from BMSCs, exosomes obtained from MSCs pre-treated with KGN exhibited enhanced chondrogenic matrix formation and reduced levels of matrix degradation in both *in vitro* and *in vivo* investigations (Liu C. et al., 2020). These findings suggest that utilizing a chondrocyte inducer like KGN may enhance the bioefficacy of exosome preparation.

Cellular autophagy, the process wherein aging organelles fuse with and are degraded by lysosomes through autophagosomes, represents the cellular mechanism through which cells utilize their components to uphold biological equilibrium. Notably, mTOR functions as a negative regulator of autophagy, and inhibiting mTOR enhances cellular autophagy, thereby contributing to the protection of articular cartilage (Caramés et al., 2012). Several studies have validated that exosomes derived from infrapatellar fat pad stem cells can confer chondroprotective and reparative effects by modulating the mTOR autophagy pathway via miR-100-5p and inducing autophagy through miR-429 targeting of fasciculation and elongation protein zeta-2 (FEZ 2) (Wu et al., 2019; Meng C. et al., 2023). To simulate the impact of exosomes released in a hypoxic microenvironment on chondrocytes, research has compared the effects of exosomes generated by ADSCs on chondrocytes under normoxic and hypoxic conditions. Results revealed that hypoxic conditions can upregulate the expression of chondrocyte proliferation genes (NDRG family member 3 [NDRG3], collagen type II alpha 1 chain [Col2a1]), while downregulating the expression of chondrocyte matrix-degrading genes, such as matrix metallopeptidase 13 (MMP-13), ultimately facilitating cartilage repair (Zhao J. et al., 2023). Additionally, ADSCs-Exos suppresses the Wnt/β-catenin pathway by targeting Wnt3 and Wnt9a with miR-376c-3p (Li F. et al., 2023), leading to the attenuation of cartilage matrix degradation and synovial membrane fibrosis. Conversely, exosomes released from ADSCs treated with protoelastin (TE) enhance chondrocyte extracellular matrix synthesis by transporting miR-451-5p, which contributes to OA progression (Meng S. et al., 2023). ADAMTS has been identified as a potential arthritis biomarker. Intriguingly, ADSCs-Exos can deliver miR-93-5p to target ADAM metallopeptidase with thrombospondin type 1 motif 9 (ADAMTS9), inhibiting the inflammatory response and decelerating cartilage damage progression (Li Y. et al., 2023).

Oxidative stress triggers inflammatory and matrix catabolic pathways in articular cartilage, contributing to cartilage damage (Chen G. et al., 2024). Additionally, members of the peroxiredoxin family exhibit protective effects against oxidative stress-induced cartilage injury. Studies have demonstrated that exosomes from adipose mesenchymal stem cells mitigate chondrocyte oxidative stress, alleviate endoplasmic reticulum stress, and boost the expression of the autophagy marker microtubule-associated protein 1 light chain 3 beta (MAP1LC3B), with peroxiredoxin 6 (Prdx6) and miR-486-5p playing pivotal roles (Guillén et al., 2021; Wang et al., 2022).

#### 4.1.3 Synovial mesenchymal stem cells -derived exosomes

Synovial mesenchymal stem cells (SMSCs) exhibit multidirectional differentiation potential and have been utilized in experimental interventions in animal models of OA (Fan et al., 2009; To et al., 2019; Wang et al., 2020; Wang et al., 2021). However, the precise mechanism of action remains to be elucidated. extracellular vesicles derived from synovial mesenchymal stem cells (SMSCs-Exos) exhibiting elevated expression of miR-302c have the capability to target ADAM metallopeptidase domain 19 (ADAM19), facilitating chondrocyte proliferation, suppressing cartilage inflammation and ECM degeneration, thereby fostering the repair of osteoarthritic cartilage (Kong et al., 2023b). Bone morphogenetic protein (BMP), a crucial growth factor within the transforming growth factor β (TGF-β) superfamily, is intricately linked to the maintenance and repair of cartilage homeostasis. Notably, SMSCs-Exos exhibiting elevated levels of bone morphogenetic protein 7 (BMP-7) facilitated the polarization of macrophages towards the M2 phenotype, leading to inflammation reduction and improvement in pathological alterations within the cartilage (Sun et al., 2023). Neurofibrillar protein-1 (NRP-1) is a transmembrane protein expressed across various tissues and has been recently implicated in the regulation of bone metabolism. Additionally, matrilin-3 (MATN-3) serves as a pivotal matrix protein secreted by chondrocytes, constituting the third member of the ECM protein family. Studies demonstrated that SMSCs-Exos upregulated miR-485-3p to suppress NRP-1 expression. Furthermore, these vesicles delivered MATN-3 to concomitantly modulate the interleukin 17a (IL-17)-mediated activation of the PI3K/AKT/mTOR pathway, resulting in the inhibition of cartilage matrix degradation (Long et al., 2023; Qiu et al., 2024). Furthermore, studies have revealed that SMSCs-Exos are enriched in miR-140-5p and miR-212-5p, which target and suppress e74 like ets transcription factor 3 (ELF3) expression, resulting in the downregulation of inflammatory mediators (interleukin 6 [IL-6], c-c motif chemokine ligand 2 [MCP-1], tumor necrosis factor α [TNF-α], cyclooxygenase-2 [COX-2], and inducible nitric oxide synthase [iNOS]). Additionally, miR-140-5p enhances chondrocyte proliferation and migration capacity without compromising the integrity of the cartilage matrix, representing a promising avenue for potential cartilage repair applications (Tao et al., 2017; Zheng et al., 2022).

#### 4.1.4 Other stem cells-derived exosomes

Human umbilical cord mesenchymal stem cells (HucMSCs) are recognized for their angiogenic and osteogenic properties (Wang et al., 2023; Yang et al., 2024). Within chondrocytes, the involvement of NADPH oxidase 4 (NOX4) in oxidative stress processes is pivotal. Studies have revealed a downregulation of miR-21-5p expression in cartilage tissues from patients with osteonecrosis of the femoral head. It was elucidated that SRY-box transcription factor 5 (SOX5) could enhance enhancer of zeste homolog 2 (EZH2) transcription, collectively counteracting the effects of miR-21-5p. Simultaneously, miR-21-5p demonstrated the ability to suppress both SOX5 and enhancer of zeste 2 polycomb repressive complex 2 subunit (EZH2) expression (Fang et al., 2022), thus fostering angiogenesis and bone repair. Moreover, the delivery of miR-100-5p and miR-23a-3p to chondrocytes targeted NOX4 and phosphatase and tensin homolog (PTEN), consequently promoting AKT expression to mitigate oxidative stress and apoptosis, thereby facilitating cartilage regeneration (Hu et al., 2020; Li X. et al., 2021). In the realm of cartilage restoration, umbilical cord MSCs and pluripotent stem cell-differentiated MSCs showcased superiority over adipose MSCs. Notably, pluripotent stem cell-differentiated MSCs exhibited greater efficacy than synovial MSCs, followed by bone marrow MSCs, in OA treatment (Zhu et al., 2017; Li Q. et al., 2021; Xu T. et al., 2024). Long non-coding RNAs (lncRNAs), acting as “molecular sponges,” can absorb specific miRNAs, thereby suppressing their target gene expression. For instance, lncRNA H19 within HucMSCs-Exos can absorb miR-29b-3p, elevate TGF-β1 and SMAD family member 3 (Smad3) levels, and inhibit forkhead box o3 (FoxO3) expression, thereby propelling cartilage repair and regeneration (Yan et al., 2021). As the exploration of various MSC types continues to expand, the potential of additional MSCs remains to be unraveled in future investigations.

### 4.2 Synovial fibroblasts-derived exosomes

Synovial fibroblasts have garnered increased attention owing to their close proximity to articular cartilage within synovial tissue, comprising macrophage-like synoviocytes and fibroblast-like synoviocytes (Damerau et al., 2024; Hu Z. et al., 2024). Exosomes derived from both cell types have been identified as contributors to the exacerbation of cartilage inflammatory responses through modulation of the TLRs/NF-κB pathway (Ma T. et al., 2024). Notably, the pronounced impact of macrophage-like synoviocytes and the ability of fibroblast-like synoviocyte exosomes to induce macrophage glycolysis activation, consequently fostering M1 cell polarization, but HIF 1α inhibited this process, highlight novel insights into the role of fibroblast-like synoviocyte-derived exosomes in inflammatory-induced cartilage damage (Liu B. et al., 2024; Wang H. et al., 2024). Distinct variations were observed in the impact of small molecule RNAs from synovial fibroblast exosomes on chondrocytes; for instance, miR-19b-3p and miR-106b were identified to exacerbate articular cartilage damage (Liu D. et al., 2020; Kong et al., 2023a), whereas miR-182-5p, miR-214-3p, miR-126-3P, and miR-142-5p exhibited potential in promoting articular cartilage repair (Zhou et al., 2021; Zeng et al., 2022; Ji et al., 2023; Lai et al., 2023). Furthermore, the enrichment of miR-106b in synovial fibroblast exosomes, targeting pyruvate dehydrogenase kinase 4 (PDK4) and modulating the RANKL/RANK/OPG pathway, underscored its role in advancing cartilage damage progression, thus identifying miR-106b as an actionable target (Liu D. et al., 2020). Moreover, the interaction between miR-106b and long non-coding RNA H19 in competition for miR-106b adsorption offers a promising avenue to reverse cartilage damage and facilitate cartilage repair (Tan et al., 2020). Iron death, characterized by redox homeostasis disruption, mitochondrial dysfunction, and iron-dependent metabolic processes, emerges as a distinctive mode of cell demise impacting cartilage inflammatory responses. Noteworthy findings indicate that miR-19b-3p, transported by fibroblast-like synoviocyte exosomes, inhibits solute carrier family seven member 11 (SLC7A11), thereby exacerbating chondrocyte iron death and cartilage damage (Kong et al., 2023a). Collectively, the adverse impact of synovial fibroblasts on cartilage repair pathways underscores the destructive consequences of inflammation on cartilage integrity, emphasizing the imperative focus on inhibiting inflammation for successful cartilage repair strategies.

### 4.3 Chondrocytes-derived exosomes

Chondrocyte-derived exosomes exhibit similarity to chondrocytes, ensuring a more reliable safety profile (Chen J. et al., 2024). Regenerated cartilage from BMSCs-Exos displays a fibrous nature, in contrast to the hyaline cartilage formation facilitated by chondrocyte-derived exosomes, maintaining chondrocyte phenotype integrity over an extended period (Chen J. et al., 2024). However, exosomes derived from degenerative chondrocytes have the potential to hasten the process of cartilage calcification (Liu Q. et al., 2022). Notably, a study investigating exosomes from osteoarthritic chondrocytes revealed their ability to inhibit autophagy related 4B cysteine peptidase (ATG4B) expression via miR-449a-5p, impeding macrophage autophagy and leading to reactive oxygen species (ROS) accumulation. This suggests a possible exacerbation of OA progression by miR-449a-5p, underscoring the significance of chondrocyte physiological conditions in determining the efficacy of miR-449a-5p for cartilage repair (Ni et al., 2019).

Research has elucidated the mechanosensitivity of condylar cartilage, demonstrating its ability to interpret mechanical stimuli into biochemical signals (Zhao et al., 2017). Furthermore, examination of individual chondrocyte cells from distinct weight-bearing zones within the tibial plateau has unveiled differential gene expression patterns in response to varying mechanical loads, emphasizing the importance of studies on chondrocytes under differing stress conditions (Wang J. et al., 2024). Circular RNAs (circRNAs) have emerged as key players in OA development, with a specific focus on circ-PRKCH, which exhibits significant involvement in disease progression. Elevated levels of Circ-PRKCH and ADAM metallopeptidase with thrombospondin type 1 motif 5 (ADAMTS 5) were detected in cartilage from arthritis patients, concomitant with decreased miR-502-5p expression. Subsequent investigations unveiled that Circ-PRKCH sequesters miR-502-5p, modulating ADAMTS 5 expression to drive inflammatory processes in articular cartilage. Additionally, interleukin 1 beta (IL-1β)-treated chondrocytes were found capable of transporting circ-PRKCH via exosomes, hinting at its potential as a biomarker (Liu Z. et al., 2022). Conversely, another study reported upregulation of Circ_0001846 and WNT 5B in OA patients and IL-1β-treated chondrocytes, accompanied by decreased miR-149-5p levels. Subsequent analyses demonstrated that silencing of Circ_0001846 reversed IL-1β-induced proliferation, apoptosis, migration, inflammation, and extracellular matrix degradation in chondrocytes. Furthermore, miR-149-5p hindered these processes by targeting WNT 5B. These results suggest that Circ_0001846, originating from chondrocytes in OA patients, sequesters miR-149-5p, subsequently disrupting its involvement in the WNT 5B axis and compromising its chondroprotective effects, thereby contributing to cartilage damage (Zhu et al., 2021), offering a promising avenue for potential OA therapies.

### 4.4 Macrophages-derived exosomes

**FIGURE 2:**
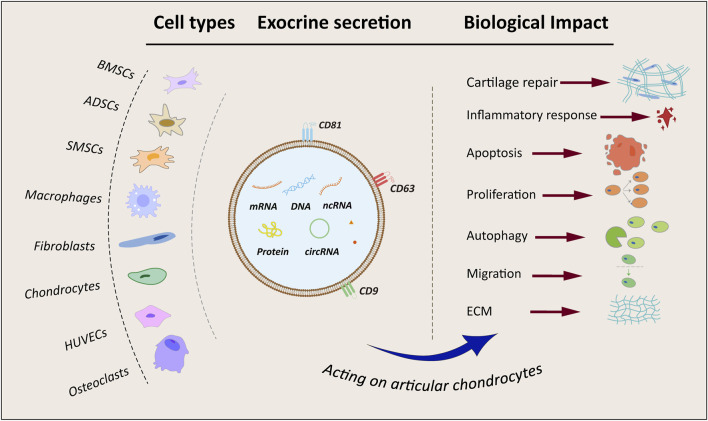
Flowchart and summary of exosomes from different sources. BMSCs, bone marrow mesenchymal stem cells; ADSCs, adipose-derived mesenchymal stem cells; SMSCs, synovial mesenchymal stem cells; HUVECs, human umbilical vein endothelial cells; ECM, extracellular matrix; mRNA, coding RNA; DNA, deoxyribonucleic acid; ncRNA, non-coding RNA; circRNA, circular RNA.

Macrophages, pivotal cells in bone immunity, undergo differentiation into pro-inflammatory M1 macrophages and anti-inflammatory M2 macrophages (Hu K. et al., 2023), crucial for regulating bone metabolism and maintaining bone homeostasis. Research demonstrated that exosomes derived from M1 macrophages upregulated the expression of interleukin 6 (IL-6), interleukin-8 (IL-8), matrix metallopeptidase 1 (MMP1), matrix metallopeptidase 3 (MMP3), matrix metallopeptidase 9 (MMP9), and matrix metallopeptidase 13 (MMP13) in chondrocytes. Subsequent investigations indicated that this impact was intricately linked to the suppression of glycogen synthase kinase 3 beta (GSK3β) and axis inhibition protein 2 (Axin2) expression by miR-1246, ultimately activating the Wnt/β-catenin pathway. Thus, this elucidated the mechanism by which M1 macrophage exocytosis exacerbates cartilage damage (Zhang et al., 2024). Furthermore, M2 macrophages perform a critical anti-inflammatory function during the advancement of OA. Studies indicated that exocytosis from M2 macrophages downregulates the PI3K-Akt-mTOR pathway, leading to the suppression of IL-1β, IL-6, and TNF-α expression, ultimately promoting cartilage restoration (Da-Wa et al., 2021). Moreover, by sequencing M1 and M2 macrophage exosomes, miR-26b-5p and miR-127-3p were significantly upregulated in M2-Exos, while miR-134-5p was significantly downregulated. Validation of miR-26b-5p *in vitro* showed that it inhibited toll like receptor 3 (TLR3) expression, thereby preventing chondrocyte hypertrophy, promoting the differentiation of M1-type macrophages to M2-type macrophages, and suppressing inflammatory responses (Qian et al., 2024). Stimulating macrophage polarization from pro-inflammatory M1 towards anti-inflammatory M2 using exosomes could represent a promising therapeutic strategy for OA management. A flowchart depicting exosome origins and altered cartilage biology is shown in Figure 2.

### 4.5 Herbal medicine-derived EVs

The secretion of extracellular vesicles (EVs) by plant cells has long been a subject of scrutiny owing to the presence of their robust cell walls. Nevertheless, advancements in research methodologies have increasingly unveiled compelling evidence supporting the capacity of plant cells to discharge plant-derived vesicles (PDVs) (Regente et al., 2009; Liu N. J. et al., 2020; Niu et al., 2023). These PDVs, characterized as intricate exosome-like structures with diverse and multifunctional traits, exhibit exceptional absorption capabilities in organisms (Garaeva et al., 2021). This property not only mitigates the longstanding challenge of limited bioavailability of active plant constituents but also opens avenues for the utilization and development of extracellular lipid nanoparticles (ELNs) as biotherapeutic agents and carriers for drug delivery (Cong et al., 2022; Zhao Y. et al., 2023). In recent times, there has been a surge in reports focusing on the use of herbal remedies for the management of bone and joint disorders. Contemporary studies have demonstrated that bioactive compounds extracted from medicinal herbs contain active ingredients that confer anti-inflammatory, anti-apoptotic, and anti-oxidative stress effects (Li and Zhang, 2020). The methodology for isolating vesicles rich in biologically potent substances from herbal remedies has reached a state of maturity, offering a novel perspective for the prospective application of herbal medications in addressing bone and joint ailments (Liu Y. et al., 2024). Notably, *Morinda Officinalis* encompasses a diverse range of anti-osteoporosis active constituents, and extracellular vesicles obtained from *Morinda Officinalis* exhibit the ability to stimulate osteoblast proliferation and differentiation via the mitogen-activated protein kinase (MAPK) pathway (Cao et al., 2024). Furthermore, extracellular vesicles sourced from *Rhizoma Drynariae* can modulate the ER-alpha (ERα) pathway to promote the differentiation of BMSCs into osteoblasts for the management of orthopaedic conditions (Zhao et al., 2024). Collectively, these investigations suggest that extracellular vesicles derived from herbs present a promising avenue for the effective treatment of OA.

### 4.6 Other types of cells -derived exosomes

Exosomes originate from a diverse array of sources, among which are exosomes derived from osteoclasts that likely play a pivotal role in modulating bone metabolism (Stegen and Carmeliet, 2024). Research has indicated that exosomes originating from osteoclasts have the capability to modulate the transforming growth factor beta 1 (TGF-β1)/SMAD family member 2 (Smad2) signaling pathway through miR-212-3p, thereby expediting the degradation of cartilage matrix. This highlights the potential benefit of suppressing miR-212-3p expression to facilitate cartilage repair, while also identifying the TGF-β1/Smad 2 axis as a plausible target for enhancing cartilage restoration (Dai et al., 2024). Furthermore, exosomes released by osteoclasts have been implicated in hastening the progression of OA. Conversely, the downregulation of RAB27A, member RAS oncogene family (Rab 27a), achieved through the inhibition of key miRNA processing enzymes or small interfering RNAs, results in diminished exosome secretion from osteoclasts, subsequently mitigating the advancement of OA (Liu et al., 2021).

**FIGURE 3:**
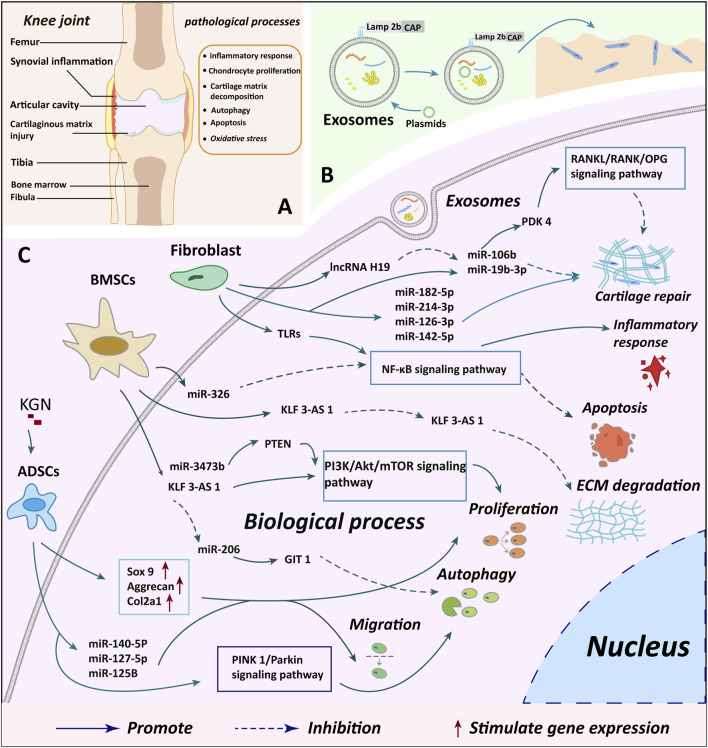
Summary of cartilage microenvironment regulation and cartilage repair mechanisms by different cellular exosomes. **(A)** A depicts the observed changes in injury and knee joint structure in knee OA cases, offering insights into the structural impacts of the condition **(B)** B illustrates the process of plasmid transfection of exosomes and genetic modification to enhance the targeting of exosomes, indicating potential avenues for novel treatment approaches **(C)** C delineates the specific molecular mechanisms through which exosomes from various cellular origins exert their biological effects on chondrocytes, providing a detailed understanding of the biological interactions involved in the disease. BMSCs, bone marrow mesenchymal stem cells; ADSCs, adipose mesenchymal stem cells; KGN, kartogenin; miR, microRNA.

Excessive free radicals in the joints trigger oxidative stress, posing a risk for the degradation of the cartilage matrix and the progression of OA (Da et al., 2024). Exosomes extracted from human umbilical vein endothelial cells (HUVEC-Exos) intriguingly stimulated the production of reactive oxygen species (ROS) in chondrocytes. Additionally, they suppressed autophagy and P21 expression, diminishing chondrocyte resilience to oxidative stress, consequently fostering chondrocyte apoptosis, which hampers the repair of OA cartilage (Yang et al., 2021). This observation suggests that the vasculature has an adverse impact on cartilage repair. Inhibiting exosome secretion from intra-articular vascular endothelial cells could potentially serve as a therapeutic approach. Due to its close proximity to the cartilage, the articular subchondral bone plays a pivotal role in the investigation of cartilage repair (Hu Y. J. et al., 2024). Studies have demonstrated a close association between exosomes originating from osteoblasts in the articular subchondral bone and cartilage degeneration. Subsequent investigations unveiled that this mechanism is linked to miR-210-5p, and inhibiting miR-210-5p substantially resulted in chondroprotective effects (Wu X. et al., 2021). Table 1 and Figure 3 depict a summary of the mechanisms by which exosomes from various cell types impact cartilage repair.

**TABLE 1:** Mechanism of different cell-derived exosomes in the cartilage repair process of osteoarthritis.

Source of EVs	miRNA	Signal pathway	Mechanism of action	References
BMSCs	KLF 3-AS 1 adsorbs miR-206	PI3K/Akt/mTOR	Promote chondrocyte proliferation; inhibit chondrocyte apoptosis	Liu et al. (2018a), Wen et al. (2022)
	miR-3473b	PTEN/AKT		Liu et al. (2018b)
	miR-326	STAT1/NF-κB/p65	Inhibit chondrocyte apoptosis	Xu and Xu (2021)
	miR-210	NF-κB	Inhibit inflammatory response	He et al. (2020)
ADSCs	miR-100-5p	mTOR	Promote chondrocyte autophagy	Wu et al. (2019)
	miR-429	FEZ 2	Inhibit chondrocyte degradation; inhibit synovial fibrosis	Meng S. et al. (2023)
	miR-376c-3p	Wnt/β-catenin		Li F. et al. (2023)
	miR-93-5p	ADAMTS9	Inhibit inflammatory response	Li Y.et al. (2023)
SMSCs	miR-485-3p	PI3K/AKT/mTOR	Inhibit cartilage matrix degradation	Long et al. (2023), Qiu et al. (2024)
	miR-140-5p		Promote chondrocyte proliferation and migration; protect cartilage matrix	Tao et al. (2017)
	miR-212-5p	ELF3	Inhibit inflammatory response	Zheng et al. (2022)
	miR-302c	ADAM19	Promote chondrocyte proliferation; inhibit inflammation; inhibit ECM degradation	Kong et al. (2023b)
HucMSCs	miR-21-5p		Promote angiogenesis; promote bone repair	Fang et al. (2022)
	miR-100-5p	NOX 4	Inhibit oxidative stress in chondrocytes; inhibit apoptosis	Li X. et al. (2021)
	miR-23a-3p	PTEN/AKT		Hu et al. (2020)
	LncRNA H19	Adsorb miR-29b-3p	Enhance chondrocyte activity; promote chondrocyte migration; promote cartilage matrix formation; inhibit chondrocyte apoptosis and senescence	Yan et al. (2021)
Fibroblast	lncRNA H19	Adsorb miR-106b; RANKL/RANK/OPG	Inhibit cartilage matrix degradation; promote chondrocyte proliferation and migration	Tan et al. (2020)
Chondrocyte	miR-214-3p	ATF 7/TLR 4; RUNX 1/VEGFA	Promote M2 macrophage polarisation; attenuate inflammatory response; promote angiogenesis	Lan et al. (2023)
	RNACirc_0001846	Adsorb miR-149-5p/WNT	Promote chondrocyte proliferation and migration; inhibit chondrocyte apoptosis; inhibit cartilage matrix degradation; inhibit inflammatory response	Zhu et al. (2021)
M2 macrophage	miR-26b-5p	TLR 3	Inhibit chondrocyte hypertrophy; promote M1 to M2 differentiation; inhibit inflammatory response	Qian et al. (2024)
Osteoclast	miR-212-3p	TGF-β1/Smad 2	Accelerate cartilage matrix degradation	Dai et al. (2024)

BMSCs, bone marrow mesenchymal stem cells; ADSCs, adipose mesenchymal stem cells; SMSCs, synovial mesenchymal stem cells; HucMSCs, human umbilical cord mesenchymal stem cells; EVs, extracellular vesicles; PI3K/Akt/mTOR, phosphoinositide 3-kinase/protein kinase b/mammalian target of rapamycin; PTEN/AKT, phosphatase and tensin homologue deleted fromchromosome 10/protein kinase b; STAT1/NF-κB/p65, signal transducer and activator of transcription 1/nuclear factor kappa b subunit 1/p65-relA; FEZ 2, fasciculation and elongation protein zeta 2; Wnt, wingless; ADAMTS9, ADAM metallopeptidase with thrombospondin type 1 motif 9; ELF3, e74 like ETS transcription factor 3; ADAM19, ADAM metallopeptidase domain 19; NOX 4, NADPH oxidase 4; RANKL/RANK/OPG, TNF-related activation-induced cytokine/TNF receptor superfamily member 11a/TNF receptor superfamily member 11b; ATF 7/TLR 4, activating transcription factor 7/toll like receptor 4; RUNX 1/VEGFA, runt-related transcription factor 1/vascular endothelial growth factor A; TLR 3, toll like receptor 3; TGF-β1/Smad 2, transforming growth factor beta 1/mothers against decapentaplegic homolog 2; miRNA, microRNA.

## 5 Engineered exosomes

Exosomes present a promising avenue for OA treatment (Xavier et al., 2023); however, they rely on specific matrix components to ensure enduring repair. Scaffold materials utilized in musculoskeletal disorders encompass organic elements (e.g., fibrin, hyaluronic acid, chitosan, collagen, alginate, and silk fibroin), inorganic compounds (e.g., hydroxyapatite, tricalcium phosphate, glass ceramics, and titanium), and biodegradable substances (e.g., polycaprolactone, polylactic acid [PLA], polyglycolic acid, and polylactic-co-glycolic acid [PLGA]) (Wu N. et al., 2021; Rahman et al., 2022; Zhang et al., 2023; Yuan et al., 2024). Furthermore, 3D cell culture systems have undergone comprehensive investigation in current studies (Lee and Lee, 2022). The 3D system’s capability to culture a higher number of cells concurrently for increased exosome production, in conjunction with the hypoxia-induced cell aggregation that stimulates cells to produce growth factors, has proven advantageous in fostering cartilage repair (Duan et al., 2022). *In vitro* studies demonstrated that 3D-Exos exhibited enhanced capacity in regulating the microenvironment of the joint cavity, with sequencing revealing its association with elevated miRNA expression; however, further investigation is required to elucidate the precise molecular mechanism (Yan et al., 2023).

To enhance the sustained release effect of exosomes, a study combined gelatin hydrogel with BMSCs-Exos, demonstrating its ability to maintain release for 14 days with an exosome release rate of 80%. This combination was shown to modulate immunity to support the repair of damaged cartilage, indicating a significant potential for the application of biomaterials and extracellular vesicles (Guan et al., 2022a). The advent of CRISPR technology in 2012 has offered promise for disease treatment. By incorporating chondrocyte affinity peptide (CAP) at the N-terminus of the exosome surface protein Lamp 2b to create exosomes targeted at chondrocytes, the efficiency of biosignalling within the exosome can be significantly enhanced, improving the exosome’s targeting capabilities (Liang et al., 2022). Lithium-containing scaffolds have been shown to effectively enhance cartilage regeneration. Treatment of BMSC-derived exosomes with lithium-containing blocking glass ceramics (Li-BGC) can employ miR-455-3p to inhibit histone deacetylase 2 (HDAC2) and enhance histone H3 acetylation, thereby promoting cartilage regrowth. This validates the critical role of lithium ions in promoting cartilage formation (Liu et al., 2023). To mimic the normal anatomical structure of cartilage, a double-layer gel was utilized to simulate cartilage and subchondral bone matrix components, combined with cartilage-derived exosomes. The results indicate an enhancement in chondrocyte migration ability, contributing to articular cartilage repair (Nikhil and Kumar, 2022). Chondroitin sulfate, a glycosaminoglycan secreted by chondrocytes, promotes cartilage regeneration and pain relief. By combining exosome carriers with pro-cartilage repair cytokines like chondroitin sulfate, not only is the release of exosomes promoted, but it also aids in cartilage matrix formation in a drug-delivery manner, providing valuable insights for the design and development of exosome carriers (Guan et al., 2022; Zhang et al., 2021). These findings suggest that incorporating cartilage repair-related cytokines into exosome-carrying hydrogels shows promising applications for cartilage repair.

## 6 Discussion

Given that chondrocytes are the sole cell type found within cartilage, this review centers its attention on the impact of various cellular exosomes on chondrocytes. The influence of exosomes derived from different cellular origins on regulating the OA cartilage microenvironment and aiding in cartilage repair involves the modulation of multiple essential pathways and downstream genes. These effects significantly alter the biological functions of chondrocytes, particularly in terms of suppressing inflammation, remodeling the cartilage matrix, enhancing chondrocyte proliferation, regulating autophagy and apoptosis, promoting directed migration to injury sites, influencing mitochondrial metabolism, and reducing oxidative stress. Engineered exosomes have demonstrated enhanced precision in targeting and more pronounced interventional effects, representing a pivotal technology for advancing clinical investigations related to exosomes. However, there remains a scarcity of studies on the differences in the efficacy of exosomes in facilitating cartilage repair across various cell types. It is crucial to identify the optimal donor cells for exosomes and explore potential synergistic or antagonistic effects among exosomes released by diverse cell sources.

While the role of pertinent signaling molecules in exosomes for promoting cartilage repair is extensively demonstrated, existing studies have primarily focused on both cellular and animal models. Notably, there is a lack of clinical trials assessing the use of exosomes for OA treatment. Treatment with MSC-derived exosomes (MSC-Exos) has demonstrated significant preclinical efficacy in animal models of OA (Zhou et al., 2022). Although clinical reports on the use of MSC-Exos in osteoarthritis patients are limited, cases involving graft-versus-host disease (Kordelas et al., 2014) and chronic kidney disease (Nassar et al., 2016) indicate good tolerability following systemic administration of exosomes. This suggests that clinical studies on exosomes are still in the exploratory phase but hold positive implications for future research. Developing highly standardized manufacturing protocols for the production of clinical-grade exosome products with essential bioactivities presents a significant challenge for clinical research groups. Figueroa-Valdés et al. (2025) pioneered the standardized development and intra-articular validation of clinical-grade extracellular vesicles derived from human umbilical cord mesenchymal stem cells (HucMSCs). Consistency in exosome production was achieved through the establishment of a standardized production protocol. The potential for standardized production of exosomes for clinical translation can be further validated by identifying intra-exosomal miRNA and protein profiles. The efficacy and safety of HucMSCs-Exos were further validated through *in vitro* cell and animal studies. Finally, twelve patients with moderate knee OA received a single dose of HucMSCs-Exos *via* intra-articular injection and were followed for 12 months. The results indicated no adverse effects and demonstrated significant, long-lasting improvements in pain and dysfunction throughout the 12-month follow-up period. Imaging studies revealed that the administration of HucMSCs-Exos via intra-articular injection in OA patients did not result in structural joint destruction nor promote the progression of cartilage damage. This clinical study validates, for the first time, the initial safety of exosome therapy in knee osteoarthritis (KOA) and has significant implications for future clinical studies involving exosomes.

Consequently, exploring the efficacy and safety of exosomes holds critical clinical importance. However, before their practical application, it is imperative to address potential safety risks, ensure exosome purity, and optimize their yield. Therefore, further investigation into suitable scaffolds, safer and more effective bioactive factors, utilization of plant and animal sources, and exploration of genetic modification techniques is essential to establish more favorable conditions for exosomes, validating their safety and efficacy in human contexts. Despite the challenges, the ongoing research progress signifies the increasingly vital role of exosomes in OA prevention and treatment, with exosome therapy poised to emerge as a key option for early OA treatment and prevention.
